# Systematic Evaluation of Ultrasonic In-Line Inspection Techniques for Oil and Gas Pipeline Defects Based on Bibliometric Analysis

**DOI:** 10.3390/s24092699

**Published:** 2024-04-24

**Authors:** Jie Huang, Pengchao Chen, Rui Li, Kuan Fu, Yanan Wang, Jinyao Duan, Zhenlin Li

**Affiliations:** 1 College of Mechanical and Storage and Transportation Engineering, China University of Petroleum (Beijing), Beijing 102249, China; 2022315301@student.cup.edu.cn; 2 General Research Institute, China Oil & Gas Pipeline Network Corporation, Langfang 065000, China; chenpc@petrochina.com.cn (P.C.); kjlirui@petrochina.com.cn (R.L.); fukuan@pipechina.com.cn (K.F.); wangyn07@pipechina.com.cn (Y.W.); duanjy01@pipechina.com.cn (J.D.)

**Keywords:** oil and gas, pipeline, in-line inspection, ultrasonic testing, bibliometrics, defects

## Abstract

The global reliance on oil and gas pipelines for energy transportation is increasing. As the pioneering review in the field of ultrasonic defect detection for oil and gas pipelines based on bibliometric methods, this study employs visual analysis to identify the most influential countries, academic institutions, and journals in this domain. Through cluster analysis, it determines the primary trends, research hotspots, and future directions in this critical field. Starting from the current global industrial ultrasonic in-line inspection (ILI) detection level, this paper provides a flowchart for selecting detection methods and a table for defect comparison, detailing the comparative performance limits of different detection devices. It offers a comprehensive perspective on the latest ultrasonic pipeline detection technology from laboratory experiments to industrial practice.

## 1. Introduction

Oil and natural gas account for 57.5% of the global primary energy consumption [1]. Pipelines, integral to the transportation of these resources, are distinguished by their capacity for transporting large volumes over long distances with minimal energy loss [2,3,4,5]. They are crucial in connecting the upstream and downstream sectors of the oil and gas industry and are essential in long-distance transportation. By the end of 2022, the total operational mileage of global oil and gas pipelines was approximately 2 million km, with an additional 26,708 km under construction, and it is projected to reach 2.2 million km by 2025 [6].

Data from the Pipeline and Hazardous Materials Safety Administration (PHMSA) of the United States Department of Transportation reveal that between 2003 and 2022, there were 12,785 significant pipeline incidents in the United States. These incidents resulted in 274 deaths and 1120 injuries. The average cost of each incident was around 541 million USD, leading to a total loss of 10.8 billion USD [7]. This underscores the growing need for enhanced inspection of oil and gas pipelines to prevent severe accidents, such as leaks and explosions, which can lead to environmental pollution and human casualties.

Pipeline in-line inspection (ILI) commonly employs technologies such as magnetic flux leakage (MFL), eddy current testing (EC), and ultrasonic testing (UT) [8,9,10,11]. As a prevalent and reliable method, magneto-electric composite internal inspection combines MFL and EC and dominates about 90% of the inspection market. Despite its widespread use, this method faces challenges like structural complexity (with some sections weighing up to 4 tons), limited effectiveness in detecting planar defects (particularly at girth welds), and sensitivity to inspection speed and lift-off distance. In contrast, ultrasonic testing, a wave-based method, is notable for its strong directionality and penetration power, making it more effective for identifying pipeline wall defects [12]. However, existing literature reviews generally discuss UT as one of the non-destructive testing techniques for oil and gas pipelines. For instance, Feng et al. integrate practical inspection data to elucidate the application of both conventional ultrasonics and electromagnetic ultrasonics in the detection of circumferential welds in oil and gas pipelines from the perspectives of mechanism, quantitative methods, and inspection reliability [11,13,14]. Alternatively, focusing on specific aspects of ultrasonics, researchers like Zang et al. delve into issues such as dispersion, multimode propagation, and attenuation in ultrasonic-guided wave technology. Methods to address these challenges from a theoretical standpoint are proposed, with a comprehensive overview provided of guided wave excitation devices [15]. Andika, on the other hand, outlines the application of machine learning signal processing methods in handling the high-volume, high-velocity, and diverse data generated using ultrasonic in-line inspection (ILI). This includes preprocessing, learning algorithms, outputs, and evaluation metrics [16,17]. There is a lack of systematic discussion that spans from a macro perspective (i.e., integrating practical industrial inspection scenarios with a vertical analysis of existing inspection technologies for oil and gas pipelines) to a meso perspective (i.e., a horizontal comparative analysis of ultrasonic testing technologies and the internal connections between detection methods and technologies) and down to a micro perspective (i.e., ultrasonic data analysis and signal processing). Bibliometric methods, based on clustering algorithms, offer an effective means to avoid subjective biases in literature selection [1,18,19,20]. Research indicates that there is currently no comprehensive review of ultrasonic internal inspection for oil and gas pipelines based on bibliometric methods.

The remainder of this paper is structured as follows. Section 2 introduces the data sources and research methods used in this study, detailing the primary process of literature analysis. Section 3, utilizing visualization analysis techniques, provides an overview of renowned research institutions and journals in the field of ultrasonic inspection of oil and gas pipelines worldwide. In the Section 5, a systematic review of the current research hotspots in the field of ultrasonic testing is conducted based on the results of literature clustering analysis. Starting from the current global industrial level of ultrasonic in-line inspection (ILI) detection, a flowchart for selecting detection methods and a table comparing detectable defects are provided. Furthermore, a detailed comparison of the performance limits of different detection devices is presented. The latest technologies in ultrasonic pipeline inspection are discussed from a holistic perspective, spanning from laboratory experimentation to industrial implementation. Finally, Section 6 summarizes and suggests recommendations for future research directions.

## 2. Research Methodology and Data Analyses

To accurately, objectively, and comprehensively reveal the research achievements in ultrasonic defect detection for oil and gas pipelines, this study employs data from the Web of Science (WOS) Core Collection, specifically the Science Citation Index Expanded and the Social Sciences Citation Index. The initial information retrieval was conducted using the search strategy designed in the Web of Science Advanced Search: (((TS = (ultraso* AND (test* OR inspect* OR detect*))) AND TS = (pipe* OR tube)) AND TS = (defect* OR flaw* OR CRACK OR SCC OR discontinue)) AND TS = (medic* OR patient* OR child* OR parent* OR women), resulting in the collection of 782 papers. The use of an asterisk “*” after keywords indicates multiple similar words with the same prefix (e.g., “pipe*” represents “pipe”, “pipeline”, etc.), expanding the search scope to encompass as many articles related to the research topic as possible.

**Figure 1:**
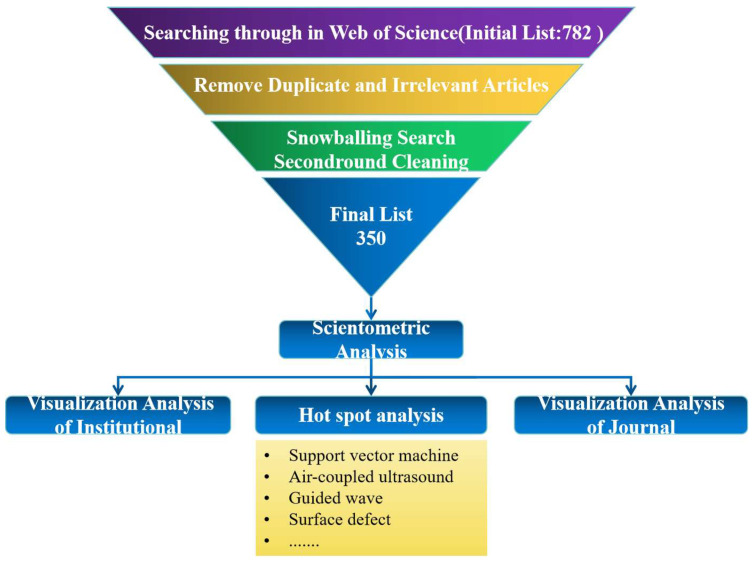
Dataset Creation Process and Research Content.

Due to potential algorithmic limitations of the WOS, the dataset may include literature irrelevant to the central theme. To address that, a rigorous screening process was applied, involving the review of titles and abstracts, and extending to full articles, when necessary, to filter out duplicates and non-relevant papers. Additionally, a snowball search method was employed to expand the dataset, which was then subjected to a second round of screening. Our research indicates that ultrasonic defect detection in oil and gas pipelines emerged as a significant area of study since the 1990s. Consequently, this paper focuses on papers, conference proceedings, and reviews published from 1992 onwards, totaling 350 documents for bibliometric analysis. The process of dataset creation and the structure of this paper are depicted in Figure 1.

After finalizing the data collection, the dataset underwent a bibliometric analysis using scientific mapping tools, specifically VOSviewer 1.6.15 and CiteSpace 6.1.R2. The analysis outcomes facilitated a detailed review of the critical research areas in ultrasonic testing for oil and gas pipelines.

## 3. Visualization Analysis

### 3.1. Visualization Analysis of Institutions and Countries

In the macroscopic research system, scientific institutions represent the smallest unit and the primary executors of research activities. To a certain extent, the number of such institutions indicates the countries leading in the field. Table 1 displays a ranking of the top ten countries globally in the ultrasonic field, sorted based on the quantity of their research institutions.

China, the UK, and Germany occupy the top three positions on the list. To ensure objectivity in ranking, additional indicators such as ‘burst’ and ‘centrality’ were also considered. “Burst”, a feature in CiteSpace, detects sudden changes in information and, when combined with the number of authors at the macro level of a country, effectively reveals the regional development level in the field. This sudden change indicates the following:

- During this period, institutions from the country experienced a sharp increase in citation frequency.
- The institutions in the country have effectively addressed crucial issues in the field.

“Centrality”, on the other hand, represents the role of a node as a bridge within a network. In social networks, centrality is used to identify “boundary spanners”. Nodes with higher centrality values play a more significant bridging role between other nodes. These institutions are highlighted with purple circles (nodes with centrality ≥ 0.1). Nodes with high intermediation centrality often connect different clustering paths and are referred to in CiteSpace as “Turning Points”.

**Figure 2:**
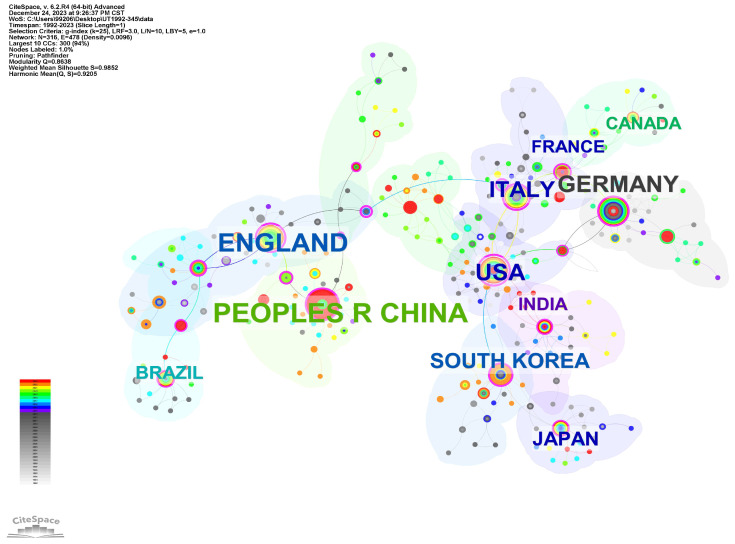
Different Country Cooperation Networks.

**Figure 3:**
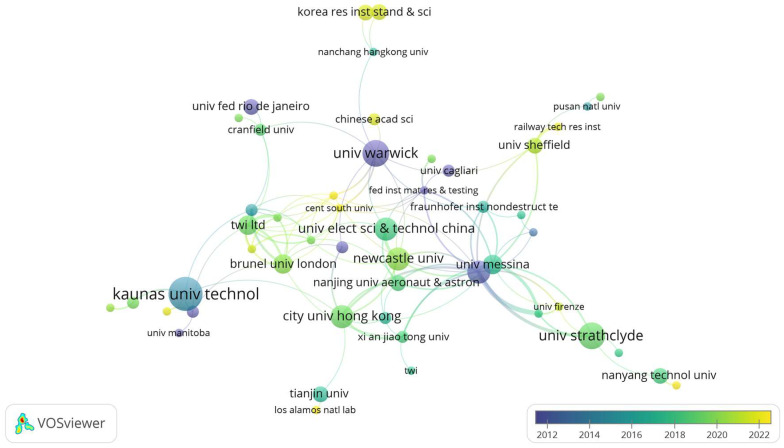
Different Institution Cooperation Networks.

In Table 2, countries in the field of ultrasonics are re-ranked from high to low based on indicators such as “Institutions”, “Burst”, and “Centrality”. By integrating data from Table 3 and Figure 2 and Figure 3, it is observed that over time, the research focus in ultrasonic testing for oil and gas pipelines has gradually shifted from Europe and America to Eurasia. The primary reason is that Europe and America, being early adopters of pipelines for oil and gas transport, have already completed the exploration phase of pipeline integrity testing. They have established reliable and comprehensive detection systems and have formally entered the phase of applying ultrasonics. In contrast, the Eurasian region is still in the nascent stages. Particularly in China, with the gradual implementation of the “High-Quality Development 2.0” and “Smart Pipeline Network” concepts, there has been a surge in demand for ultrasonic testing in pipeline inspection, spurring significant research in the field in recent years. However, from the perspective of “centrality”, the research in ultrasonic testing in the Eurasian region still lags behind the established positions of Europe and America.

### 3.2. Visualization Analysis of Journals

As illustrated, publications were visualized and analyzed using VOSviewer. In contrast to traditional metrics based solely on the number of published documents, this analysis employs “total link strength” to represent a journal’s influence. This approach provides a more comprehensive perspective for assessing journal impact, as it considers not only the volume of publications but also their inter-citation relationships. The thickness of the lines indicates the strength of the connections, while from a temporal perspective, it can signify the influence of one journal on another.

**Figure 4:**
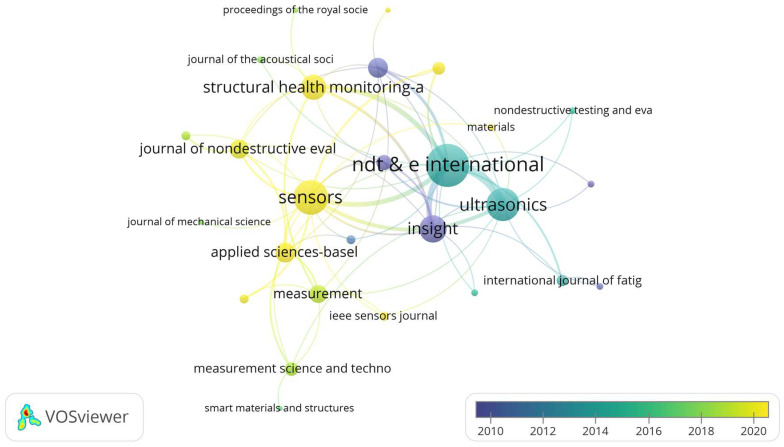
Different Journal Cooperation Networks.

Figure 4 reveals that ultrasonic testing has gradually shifted from theoretical research to practical applications over time. This transition reflects the natural progression of scientific research, moving from theoretical exploration to real-world applications. Identifying specific application scenarios for ultrasonics is becoming a research trend, suggesting that ultrasonic technology may find broader applications in the future. This is an exciting trend, as it implies that our research findings are more likely to be transformed into practical technologies and applications, bringing tangible benefits to society.

According to the data, the top ten influential journals are displayed in Table 4, with *NDT & E International*, *Sensors* and *Ultrasonics* occupying the top three positions. These journals, recognized as leading publications in the field of ultrasonics, have made significant theoretical contributions to the field of ultrasonics and influencing subsequent research.

## 4. Types of Defects and Traditional ILI Methods in Oil and Gas Pipelines

### 4.1. Common Types of Defects in Oil and Gas Pipelines

As global energy demand grows, the significance of oil and gas pipeline systems becomes increasingly pronounced [21]. The health of pipelines directly impacts the reliability and safety of the energy supply. Ultrasonic testing technology has been closely associated with concepts such as “condition assessment”, “defect detection”, and “pipeline safety” since the last century.

Defects, as the core of pipeline condition assessment, are crucial for ensuring the safety and reliable operation of oil and gas pipeline systems. Pipeline defects can be broadly categorized into three types based on their origins: manufacturing defects arising during prefabrication, welding defects and geometric defects occurring during pipeline construction, and corrosion defects formed during usage. However, the current pipeline construction quality is high with rare manufacturing defects.

Based on the shape of defects, oil and gas pipeline defects can be classified into planar defects and volumetric defects (details in Table 5).

(1) Volumetric defects encompass porosity, inclusions and corrosion, posing relatively low risks, with the primary failure mode being plastic failure.
(2) Planar defects include lack of penetration, lack of fusion, cracks, and undercutting. The defects at the root weld are the main cause of pipeline cracking and are also a focal point of research in non-destructive testing of circumferential weld defects.
(3) Structural discontinuity defects include irregular weld seam profiles, misalignment, and poor formation, leading to stress concentration and reduced material performance, further exacerbating defect propagation [22,23,24].

Corrosion in pipelines typically manifests as localized corrosion, primarily caused by pitting in buried pipelines or coating defects or dissolution. The corrosion induced by coating detachment and extensive corrosion can be precisely detected and quantified. However, pitting, as a particular case of corrosion, remains challenging in oil and gas pipeline inspection, as it can easily evolve into pinholes and lead to crack initiation, posing a higher risk of failure for high-pressure natural gas pipelines.

Pitting is a chemical process involving four stages: passive film breakdown, pit initiation, metastable pitting, and pit propagation and growth. Due to factors such as temperature, acidity, environmental conditions, and biological factors, corrosion often exhibits randomness, making it difficult to estimate corrosion rates. To address the challenge of unpredictable corrosion rates, scholars have developed corrosion rate models based on comprehensive coating information, the effectiveness of cathodic protection, specific corrosion rates of certain soils, and other pipeline characteristics. These models predict corrosion rates and depths using probability and statistical methods, including regression models and Markov process models [25].

(1) Regression models can easily estimate future corrosion defect depths in pipelines without requiring extensive mathematical knowledge for application. However, this model relies on a large amount of data and cannot account for the randomness of pitting phenomena.
(2) Markov process models consider the physical and chemical characteristics of the environment and accurately reflect the randomness of localized corrosion defect growth. However, their use requires specialized knowledge and programming skills, which may limit their widespread application.

Moreover, continuous electromagnetic in-line inspection is considered an ideal method to determine corrosion rates. However, it is challenging to determine corrosion rates using this information due to the complex pipeline environment and variations in sensor calibration methods before each inspection. Locating the same corrosion defect between two consecutive inspections may be a daunting task. It is worth mentioning that while accurate corrosion rate prediction through in-line inspection is challenging, it still provides essential information on pipeline body and wall defects required for building more accurate probabilistic and statistical models. At the same time, accurate corrosion rate models can optimize in-line inspection tasks, greatly reducing costs for pipeline operators.

### 4.2. Conventional ILI Methods for Oil and Gas Pipelines

The predominant in-line inspection methodologies in the oil and gas pipeline industry currently encompass MFL, EC and UT.

#### 4.2.1. Electromagnetic Testing

(a) Magnetic flux leakage testing

**Figure 5:**
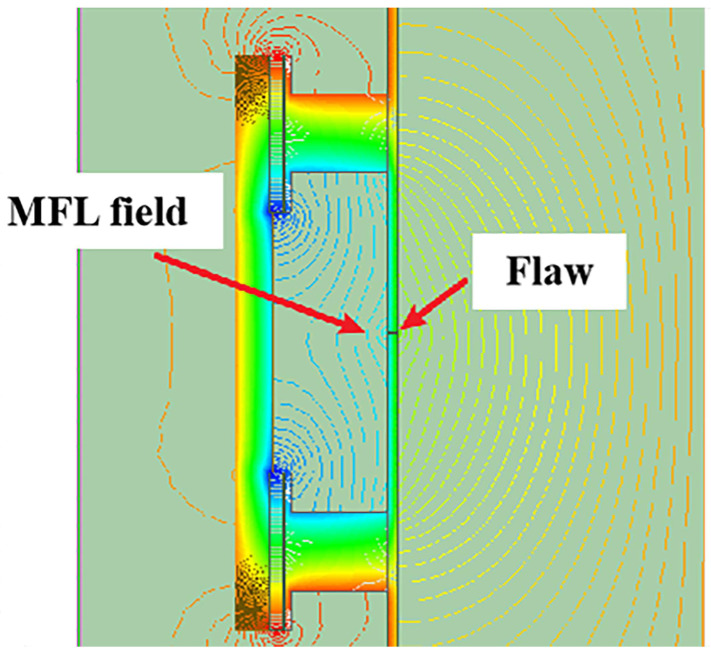
Principle of Magnetic Flux Leakage Testing.

The principle of MFL is depicted in Figure 5. Upon applying an external magnetic field that induces magnetic saturation in the pipeline, defects within the pipe wall that disrupt the magnetic flux lead to distortion and external leakage of this flux. This results in an MFL field being formed on the pipe’s surface. Triaxial Hall sensors are employed to detect this field, enabling the acquisition of detailed information regarding the defects’ distribution and dimensions [10,26,27]. MFL technology stands as a highly regarded and reliable method for pipeline inspection within the sector [28], commanding approximately 90% of the total market equipment share. Contrasting with ultrasonic testing, which is restricted to liquid pipelines, MFL is capable of detecting volumetric defects in both the interior and exterior of pipelines within a specified wall thickness limit (up to 30 mm). Furthermore, it requires less stringent cleanliness conditions for pipelines than ultrasonic testing.

**Limitations:** As depicted in Table 6, MFL exhibits a reduced efficacy in detecting planar defects, particularly at girth welds. It is notably less effective, or even incapable, of identifying narrow or closed cracks, such as those caused by stress corrosion or hydrogen-induced cracking [29,30,31].

(b) Eddy current testing

Eddy current testing operates by generating an eddy current magnetic field using an excitation coil, which interacts with the original magnetic field, thereby altering the complex impedance of the testing coil. This impedance alteration is utilized to detect and identify defects on the pipeline’s inner wall and is extensively applied in pipeline crack detection.

**Limitations:** EC’s effectiveness is limited by the skin and lift-off effects, restricting its use to the surface or near-surface inspection of conductive materials. Moreover, extracting diagnostic signals poses significant challenges and makes it difficult to accurately determine the type and size of defects [32,33,34,35]. Consequently, eddy current testing is often used in conjunction with MFL, facilitating more accurate localization of defects.

(c) Advancements in electromagnetic inspection
  (1) Advancements in Electromagnetic Inspection Equipment

In the realm of equipment, ROSEN and Baker Hughes have independently developed triaxial ultra-high-definition magnetic flux leakage internal detectors. These smart PIGs, integrated with the modules of geometric deformation and IMU, possess the capability to navigate through a diameter of 1.5 times their own and can identify open cracks exceeding 0.5 mm and pinholes of at least 5 mm, achieving resolutions of 1.6 mm circumferentially and 1 mm axially. The China Oil and Gas Pipeline Network Corporation has made significant strides in pipeline internal inspection technology, creating a triaxial ultra-high-definition electromagnetic composite internal detector specifically for 1219 mm diameter pipelines. This advanced PIG is equipped with MagEC ultra-high-definition electromagnetic composite probes, sophisticated mechanical geometric deformation sensors, and differential electromagnetic eddy current sensors. It has demonstrated a detection rate of up to 90% for pinhole defects larger than 3 mm in diameter at girth welds and 80% for open cracks that are wider than 0.5 mm, longer than 24 mm, and extend to a depth of 50% of the wall thickness.

  (2) Advancements in Electromagnetic Inspection Research

From a research perspective, the focus on electromagnetic composite detection has predominantly revolved around interpreting detection data. Cheng et al. employed the YOLOv5 and Vision Transformer (ViT) algorithms for pipeline defect inspection and classification, exploring the influence of different model architectures on defect identification performance. Comparative analysis indicates that the composite algorithm surpasses the standalone YOLOv5 algorithm in terms of accuracy in classifying pipeline defects, while maintaining its capability for high-precision defect detection [36]. Y. Shen and colleagues leveraged magnetic flux leakage (MFL) signals and convolutional neural network (CNN) technology to forecast the dimensions and locations of corrosion defects in steel pipes. Their research underscores the promising application of CNN models in enhancing pipeline integrity management [29]. Bin Liu introduced a stress factor in developing a numerical analytical model for the internal detection of complex MFL defects in pipelines. His research delved into the variations in magnetic signals induced by different sizes of defect end face segments, analyzed the patterns of MFL signal distribution under various geometric dimensions and stress conditions at the defect end faces, and evaluated their impact on the characteristics of magnetic signals [31]. Additionally, Jianhua Pan and colleagues implemented an advanced CLIQUE algorithm for marking defect regions in segmented pipelines. They then utilized the SSA_BP neural network for extracting and classifying three-dimensional MFL feature signals from these marked regions. The findings from their study reveal that this approach enhances the efficiency of defect marking and provides a more detailed analysis of the marked areas [37].

#### 4.2.2. Ultrasonic Testing

Ultrasonic testing is a technique that employs ultrasonic waves to detect macroscopic defects, characterize changes in material structure and mechanical properties, and measure the geometric features of a workpiece [38]. This method boasts advantages such as high detection speed, precision, directivity, sound wave energy, and strong penetration capability [39]. Ultrasonics, as a means of detecting metal defects, was first proposed by Sokolov of the Soviet Union in 1929, evolving subsequently into transmission testing and pulse-echo testing methods [40]. As shown in the Table 6, ultrasonic in-line testing primarily addresses issues related to pipeline cracks and metal loss. Crack detection involves the recognition and quantification of crack location and size by generating shear waves in the pipe wall using ultrasonic probes arranged at a 45° angle [41].

Currently, companies worldwide with the capability to develop complete sets of ultrasonic equipment, such as ROSEN, TDW, and China National Petroleum Corporation (CNPC), possess electromagnetic ultrasonic crack detection capabilities, with roughly equivalent detection capacities. Under speeds not exceeding 2.5 m/s, they can detect cracks as small as 50 mm in length and 2 mm in depth, with an error margin of ±1 mm in depth and ±20 mm in length. Compared to Baker Hughes and NDT Global’s UltraScan CDP/DUO and EVO Eclipse UCx, traditional ultrasonic methods using coupling agents have significant advantages in terms of detection depth and speed.

(a) Ultrasonic Phased Array

NDT Global’s PROTON, developed on the principle of ultrasonic phased array technology, employs a PAUT system to design phase delays for multiple probes. This design allows for the editing of the direction and focus of the ultrasonic beam, enabling imaging and detection at various depths and angles. It is capable of detecting not only metal corrosion and crack defects but also stress corrosion cracks, fatigue cracks, and welding crack defects [42].

(b) Time-of-Flight Diffraction (TOFD)

**Figure 6:**
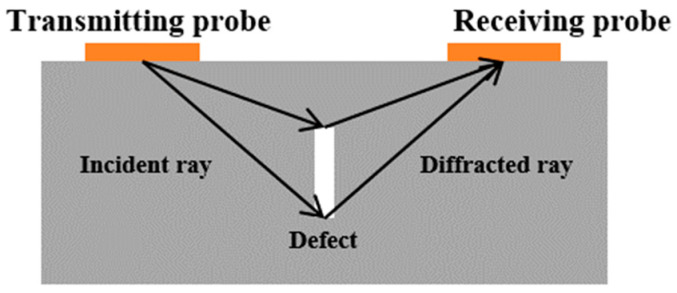
Principle of TOFD.

Another commonly used method for crack detection is the Time of Flight Diffraction (TOFD) method, first proposed by Silk in 1977 [43]. This method relies on the diffraction of ultrasonic waves by “corners” and “ends” within the internal defects, differing fundamentally from the traditional pulse-echo method, which depends on direct reflection signals [44,45,46]. As depicted in Figure 6, TOFD typically employs a dual probe arrangement with one transmitter and one receiver on the same side. This method significantly reduces the time required to scan a weld, as it does not necessitate raster scanning at each position.

(c) Ultrasonic Pulse-Echo Technique

In addition to the abovementioned applications, another form of ultrasonic testing in pipeline inspection involves emitting ultrasonic pulse waves perpendicular to the pipe wall. This method calculates the pipeline’s wall thickness by measuring the pulses’ echo times reflected off the inner and outer pipeline surfaces. This approach, introduced to the market in the early to mid-1980s, is instrumental in identifying and quantifying volumetric defects [47,48]. NDT Global’s EVO 1.0 UMp+ ultrasonic internal detector (NDT Global, Houston, TX, USA), currently deployed in petroleum pipeline inspection services, exemplifies this technology. It can quantify pipeline wall thickness and crack detection with an accuracy of ±0.4 mm when the data reliability is at 90% [49].

**Limitations:** These ultrasonic testing methods still rely on a coupling agent to address the significant impedance mismatch at the propagation interface. Oil, serving as an effective coupling agent, has facilitated the widespread application of this technology in liquid pipelines. In contrast, traditional ultrasonic testing faces challenges in natural gas pipelines, limiting its application. It is often used only as a supplementary method, performing external inspections of girth welds or spiral welds before commissioning or during the excavation verification stage, rather than being applicable for internal pipeline inspections.

### 4.3. In-Line Inspection (ILI) Tool Selection and Calibration Methods

#### 4.3.1. In-Line Inspection (ILI) Tool Selection Methods

**Figure 7:**
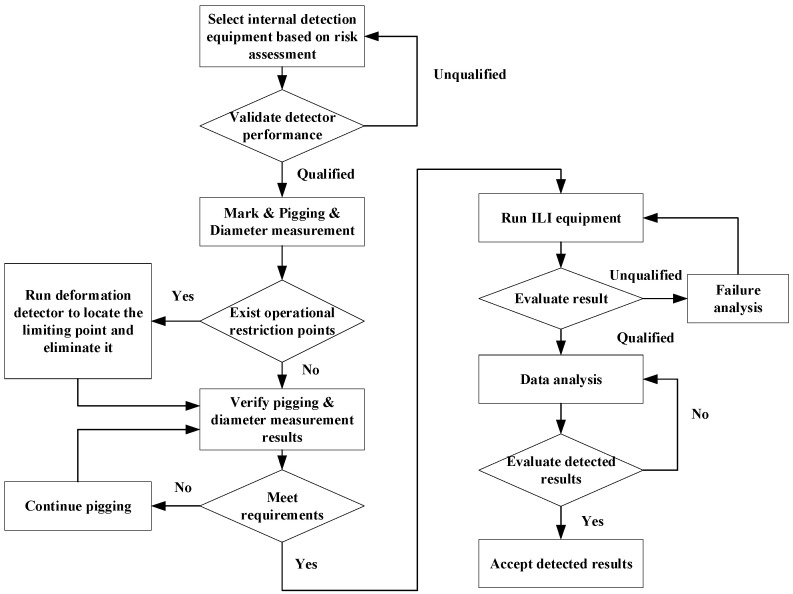
In-Line Inspection Process.

To address the inspection of different types of defects and the varying applicability of various inspection techniques, as shown in Figure 7, a pipeline risk assessment to determine suitable internal inspection techniques is conducted initially. Industrial experimental safety certification of internal inspection equipment, including but not limited to vibration, pressure resistance, strength, and detection accuracy tests, is performed to verify whether the selected detector’s performance meets the qualification requirements. Simultaneously, issues such as dents caused by improper construction and pipeline deformations caused by soil displacement significantly restrict conventional internal inspection tasks. Consequently, pigging and diameter measurement tasks are conducted two or three times before internal inspection to obtain constraint point information and carry out necessary modifications. Next, the selection of technology is conducted by assessing its suitability and capability, considering factors such as defect types, compatibility with other techniques, ease of operation, etc. Finally, an evaluation is conducted to determine the technology to be adopted, followed by data analysis. Field excavation verification is used to assess the detection quality of in-line inspection (ILI) tools, specifically their accuracy.

#### 4.3.2. In-Line Inspection (ILI) Tool Calibration Methods

As is well known, both in-line inspection (ILI) tools and field measuring instruments are subject to measurement errors, and calibration helps estimate the true magnitude of these errors. The performance of ILI tools can be assessed by statistically comparing ILI data with field excavation data to ensure the reliability of the data. It is crucial to conduct reliability and risk assessments based on the probability distribution of pipeline defects or the failure probability of individual defects. However, the calibration of ILI tools still faces challenges in the following aspects [50]:

(1) Measurement Errors: Determining the measurement errors of ILI tools and field instruments, including systematic errors (such as constant bias and non-constant bias) and random errors.
(2) Calibration Experiment: Conducting calibration experiments to compare ILI readings with field measurement depths and estimate the true size of corrosion metal loss and its associated errors.
(3) Model Verification: Checking for the presence of non-linear regression, variance heterogeneity, outliers, and non-normality of measurement errors in the model.
(4) Performance Evaluation: Evaluating the performance of ILI tools when significant measurement errors exist in field instruments and determining the number of unsuccessful field verifications.

To address these issues, scholars have proposed error assessment methods based on probability and statistics. Among them, the traditional errors-in-variables (EIV) method is used to handle cases where both measuring instruments have errors, but it requires knowledge of the variance ratio of errors or one of the variances. The Grubbs and Jaech estimators are used to estimate the variance of measurement errors, but they may produce negative variances or unreasonable variance values in practical applications. Compared to the former two, a variant method combining V-Wald and V-Jaech provides a more accurate estimation of the true defect depth and the variance of measurement errors. It distinguishes between the sampling distribution caused by non-constant bias and similar measurement tool errors. Even in cases where measurement errors are similar, it can provide reliable calibration results, which are difficult to achieve in traditional models.

## 5. Analysis of Research Hotspots

**Figure 8:**
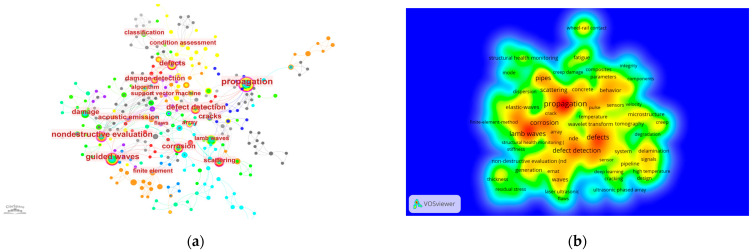
Keyword Visualization Analysis. (**a**) Citespace; (**b**) VOSviewer.

Keywords reflect the interrelationships among various themes explored in the literature, briefly summarizing the research content. Analyzing keywords is instrumental in gaining insights into the research hotspots of a field. To mitigate potential errors from using a single software, both VOSviewer 1.6.15 and CiteSpace 6.1.R2 were employed to generate the keyword co-occurrence maps shown in Figure 8a,b. These maps exhibit good consistency between the two software tools. In these maps, the size of each node or the intensity of its color represents the frequency of the keyword’s occurrence. As the frequency increases, the node’s circle enlarges, or the area’s color intensifies.

**Figure 9:**
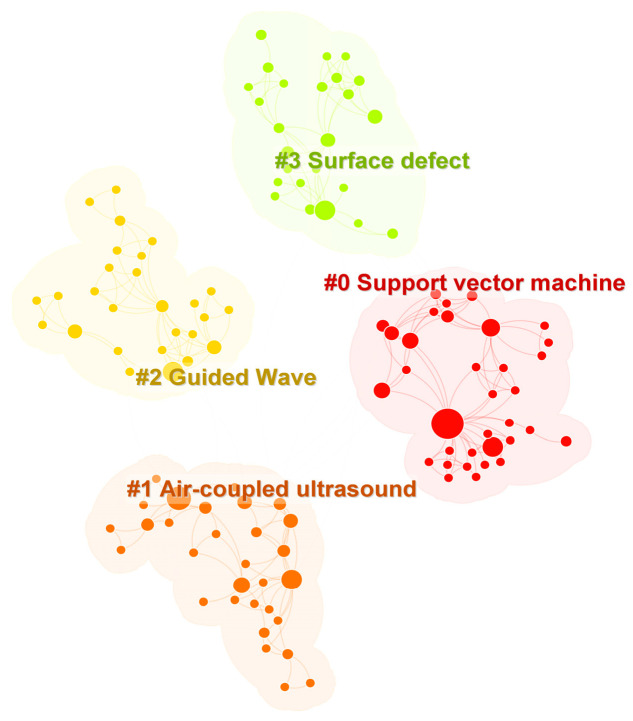
Keyword Clustering Analysis.

As demonstrated in Figure 9, cluster analysis conducted using CiteSpace 6.1.R2 identified four current research hotspots in the field of ultrasonic defect detection for long-distance oil and gas pipelines: support vector machine, air-coupled ultrasound, guided wave testing, and surface defects inspection.

**Figure 10:**
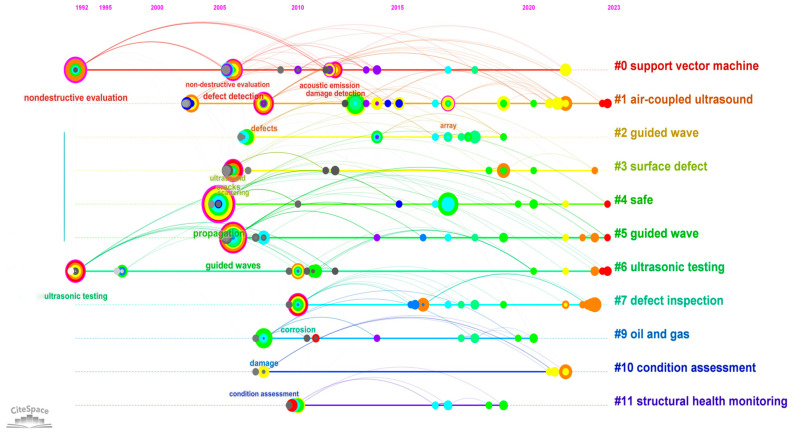
Keyword Temporal Map.

Figure 10 reveals that since 1992, ultrasonic testing has emerged as a means of non-destructive evaluation. During 2005–2015, ultrasonic technology saw widespread application in the field of defect inspection in oil and gas pipelines. However, its use in natural gas pipelines was mostly limited to external defect detection at welds during construction. In response to the limitations of magnetic flux leakage detection, particularly in identifying planar defects at girth welds, the existing methods and capabilities were found inadequate for the practical needs of the oil and gas engineering sector. This led to increased research focus on ultrasonic testing in pipelines, rapidly advancing the technology and theory of ultrasonic defect detection.

From 2015 to the present, there has been an explosive development of new acoustic testing techniques. Current ultrasonic testing in oil and gas pipelines focuses on overcoming challenges such as the inapplicability of coupling agents in natural gas pipelines (where planar defects are more problematic than in oil pipelines) and the application of artificial intelligence and data mining for the analysis and interpretation of existing ultrasonic data. This explains why concepts like support vector machine, air-coupled ultrasound, and guided waves have become hot topics in the field.

### 5.1. Surface Defect

#### 5.1.1. Conventional Surface Inspection Methods

Surface defect detection has always been a core focus of pipeline inspection. Conventional surface inspection methods include penetrant testing (PT), magnetic particle testing (MPT), and eddy current testing (as shown in Table 7). Compared to ultrasonic in-line inspection, PT and MPT offer higher sensitivity but require the removal of pipeline coatings. They also demand high cleanliness of the test surface and are not suitable for in-line inspection [3]. As mentioned in the previous section, EC has matured in internal inspection applications. However, due to the skin and lift-off effects, this technology is quite sensitive to speed and lift-off [51,52,53].

#### 5.1.2. Ultrasonic Surface Inspection Techniques

UT can effectively address the abovementioned issues. However, during the propagation of ultrasonic waves, interference can create a series of uneven sound pressure zones near the wave source, known as the near-field zone. The presence of this area can lead to inaccurate quantitative assessment of surface defects. Additionally, tiny cracks on the inner surface of the pipeline wall can easily mask larger defects below, affecting the detection of larger defects. To address this issue, scholars from around the world have conducted extensive research. Considering the near-field zone’s impact on detection, J.M. Ha et al. proposed an ultrasonic detection method based on autoencoders to inspect defects within the dead zone of the probe. This method can identify subtle deviations caused by defects. To validate the model’s effectiveness, aluminum blocks with near-surface defects were subjected to B-scan testing. The results showed that the proposed method outperforms traditional gate-based detection methods in identifying the size and location of near-surface defects [54]. Jun He and colleagues presented a quantitative approach for detecting surface anomalies using Rayleigh waves generated by lasers. Experimental results demonstrate that this method outperforms standing wave energy or reflected wave energy techniques, particularly in imaging vertical and inclined defects [55]. Similar challenges are also encountered in ultrasonic phased array testing. Tian and YK established a mathematical model combining background subtraction with the square difference algorithm based on linear acoustic theory to extract the echo features of near-surface defects. Simulation and experimental results show that this model can effectively extract the echo features of near-surface defects, achieving positioning and quantitative accuracy values of 0.2 mm and 0.3 mm, respectively, for near-surface defects [56].

### 5.2. Guided Wave Inspection Technique

**Figure 11:**
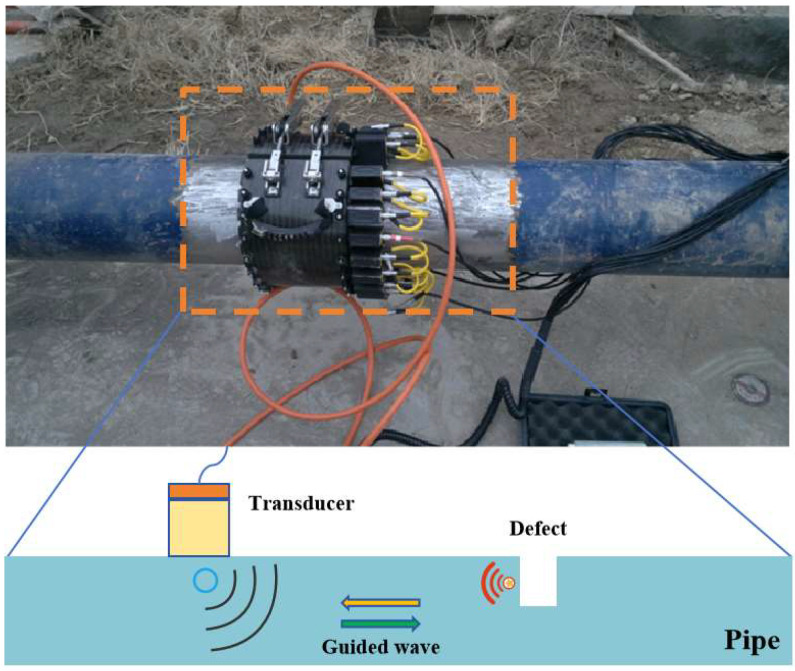
Principle of Guided Waves.

Guided wave ultrasonic testing (GWUT) is a non-destructive testing method that utilizes the propagation characteristics of ultrasonic waves in solids to detect internal structures, defects, or variations in materials [57]. Standard testing methods include “pulse-echo” and “pitch–catch” modes. Unlike bulk ultrasonic waves propagating in the direction of wall thickness, guided waves propagate axially along the pipeline, as illustrated in Figure 11. With only a single or a few probes, it is possible to effectively conduct long-distance screening of cross-sectional or axial damage in critical areas of the pipeline [58,59]. Guided wave testing, emerging as an external inspection method for non-piggable pipeline segments, began gaining prominence in the 1970s [60,61]. This technique allows for screening pipeline defects over tens of meters by merely removing a portion of the coating layer [62].

#### 5.2.1. Potential-Based Pipeline External Inspection Methods

Furthermore, there is another category of pipeline defect inspection methods based on changes in electrical potential. As Table 8 illustrates, these methods generally require manual patrolling, heavily relying on the operator’s subjective judgment. They are highly susceptible to environmental disturbances and incapable of ascertaining the state of coating delamination. Guided wave testing can detect coating delamination through changes in the dispersion characteristics of torsional waves caused by local coating [63]. However, its application in buried pipelines is severely constrained by the need for localized excavation and coating removal, limiting the widespread adoption of this technology [64].

#### 5.2.2. Non-Contact Ultrasonic-Guided Wave Testing

The advent of novel non-contact ultrasonic testing technologies has expanded the possibilities of using guided waves for internal pipeline inspection. Non-contact ultrasonics can be categorized into electromagnetic ultrasonics, laser ultrasonics, and air-coupled ultrasonics. Their principles, advantages, and limitations are shown in Table 9.

(a) Laser Ultrasonic-Guided Waves Testing

Owing to high maintenance costs and factors like surface roughness, speed, and environmental vibrations, LUGWT is predominantly used in precision industries, such as aerospace for defect detection, and remains largely experimental in pipeline applications [65]. For instance, JH et al. [55] utilized laser-generated guided waves to assess pipeline corrosion and successfully evaluated the location and size of defects in two-dimensional scan images.

(b) Electromagnetic Ultrasonic-Guided Waves Testing

In industrial in-line inspections, the applications of non-contact ultrasonic-guided waves are presently limited to EmatScan CD and ROCDeMAT-c developed by ROSEN and P II, as well as NDT Global’s ARTscan intelligent PIG as shown in Table 10. Electromagnetic ultrasonic guided waves operate through a process where alternating electric currents induce high-frequency eddy currents on the surface of a pipeline. When subjected to an external magnetic field, these eddy currents stimulate the generation of guided waves [66,67].

To deepen understanding of these technologies, scholars worldwide have conducted extensive research [68,69,70]. Nurmalia proposed an EMAT pipeline inspection technology based on high-order torsional-guided waves T(0,2), finding that phase measurement as a quantitative detection method holds considerable potential [71]. Liu introduced a novel flexible EMAT transducer for generating L(0,2)-guided waves in pipelines, demonstrating its effectiveness in corrosion detection in this mode [72]. Masahiko also developed an EMAT technique to detect corrosion defects on steel pipes’ external surface, basing axial defects’ location and depth assessment on the amplitude and phase shift responses of the round-trip signals in SH0 and SH1 modes. The results indicated that the SH1 mode is more sensitive to defects than the SH0 mode [73].

**Limitations:** In summary, non-contact ultrasonic-guided wave testing primarily utilizes Lamb and surface waves. The circumferential propagation characteristic of Lamb waves makes this technology particularly effective in detecting stress corrosion cracks in natural gas pipelines and is sensitive to crack defects at straight weld seams. However, it faces challenges in detecting minute cracks at girth welds [74,75]. Due to the multimodal nature, dispersion, and long-distance attenuation properties of ultrasonic-guided waves, the testing typically involves using broadband low-frequency modulated pulses as the excitation signal. This approach, while effective, also leads to limitations in terms of defect resolution and the accuracy of defect localization in ultrasonic-guided wave testing.

### 5.3. Air-Coupled Ultrasound

Air-coupled ultrasonics represents a novel non-contact ultrasonic testing technology, utilizing air as the coupling medium. Air-coupled ultrasonics boasts distinct features such as non-contact operation, large stand-off distance, and low power consumption. This technology is suitable for ferromagnetic materials and applies to natural gas pipelines and medium- to low-pressure pipelines where traditional ultrasonics and magnetic flux leakage methods are ineffective. Thus, it offers a broader range of applicability [76,77,78].

Air-coupled ultrasonics, compared to contact or immersion ultrasonics, experience a significant reduction in sensitivity, approximately 80 dB lower [79,80]. This energy attenuation primarily arises from three factors: ultrasonic wave attenuation in air, substantial reflection at the air–solid interface, and the efficiency of ultrasonic transducer conversion. The inherent acoustic attenuation in air and surface reflection remains an unavoidable natural phenomenon in ultrasonics, leading to low transducer efficiency and prolonged pulse reverberation due to the significant impedance mismatch.

To enhance signal quality, current research in the industry is focused on two main areas:

- Optimization of air-coupled ultrasonic probe structures and materials.

Air-coupled probes can be categorized into piezoelectric and capacitive types. Capacitive transducers operate by applying an excitation voltage between a metallized film and a conductive substrate, causing the film to vibrate under electrostatic action, thereby generating ultrasonics at specific frequencies. D.W. Schindel and others, through comparative studies, found that capacitive ultrasonic probes offer a broad frequency response and good damping, effectively addressing the issue of high central frequency present during ultrasonic wave excitation in piezoelectric ultrasonics [81]. However, due to their high cost and strong environmental dependency, capacitive probes remain largely experimental and have not yet seen widespread industrial application [82]. In 1995, Hutchins et al. achieved the fabrication of regular air layers on the conductive substrate of electrostatic transducers through etching, demonstrating these probes’ excellent bandwidth performance [83].

Advances in piezoelectric ultrasonic transducers have also been achieved, especially in their structural design. To improve resonance effects, one to three connected composite sensors have been developed, reducing the impedance of sensor materials and enhancing efficiency and coupling performance [84]. Selecting superior matching layer materials to allow more energy transmission through the air into the test object is a key factor in realizing these improvements. Alvarez-Arenas and others at the Spanish CSIC Institute of Acoustics, after researching various material characteristics, identified nylon as ideal materials (with mixed cellulose ester and polyvinylidene fluoride being suitable for frequencies above 2 MHz). This research largely resolved the issue of selecting matching materials and was the first to study the variation of material attenuation coefficients with frequency [65].

Piezoelectric probes, owing to their higher power output, have long dominated the commercial ultrasonic probe market. Systems developed by companies like Probe Corporation in Japan, and Ultran, PAC, and SONOTEC in Germany, which are based on piezoelectric transducers, are examples. Research in areas such as focused air-coupled transducers and defect detection in composite materials and lithium-ion batteries has been conducted by institutions like Beihang University, Nanjing University, and the Chinese Academy of Sciences’ Institute of Acoustics. However, there remains a lack of development in equipment specifically for internal inspection of oil and gas pipelines.

- Signal encoding

Hutchins et al. [85] utilized pulse compression technology to encode excitation signals, using capacitive sensors to generate broadband chirp signals in air for measuring solid samples. Their results demonstrated that this signal processing technique significantly enhances the signal-to-noise ratio (SNR) and detection precision of air-coupled testing, validating the feasibility of pulse compression technology in improving air-coupled ultrasonic performance. The team also conducted a comparative study of existing signal encoding techniques, concluding that the optimal choice of modulated signals depends on the available bandwidth and type of measurement [86]. Garcia and colleagues proposed using Golay sequences to encode Lamb waves excited by air-coupled ultrasonics, finding that the SNR of ultrasonic signals under Golay encoding improved by 21 dB compared to non-encoded excitation [87]. Additionally, Tang et al. introduced phase-encoded excitation and pulse compression techniques, effectively raising the SNR of received signals by over 10 dB [88]. Li and colleagues suggested using P4 polyphase sequences to encode excitation in air-coupled piezoelectric transducer-based non-destructive testing systems. Their mixed signal processing method increased the SNR by 12.11 dB and improved the time-domain resolution by approximately 35% [89].

Despite these advancements in probe design and signal encoding, the effectiveness of air-coupled ultrasonics in metal detection remains limited, particularly with high-speed operation and vibration impacts of internal detectors. Air-coupled ultrasonics was first proven in 1973 for generating Lamb waves in metal plates. Since then, Lamb waves have been extensively used in various materials, including fiber-reinforced polymer composites [90,91]. In the industrial sector, NDT GLOBAL is currently the only company applying air-coupled ultrasonic testing in the internal inspection of metal pipelines. Their developed ARTscan system (typically 400 kHz–1.2 MHz, with pressure above 7 MPa) merges low-frequency broadband signals with resonant and guided wave technologies. This not only allows for highly precise measurements of metal loss in pipelines (±0.4 mm) and the detection and classification of thick-walled defects but also facilitates comprehensive geometric measurements. This advancement represents a significant step in applying air-coupled ultrasonics in metal pipeline inspection. Compared to immersion ultrasonic, which requires coupling agents and high cleanliness, ART has a 50% deformation pass-through capability (compared to 10% for magnetic flux leakage) and 1.5D high pass-through ability, allowing for pipeline inspection without pigging. It means that when inspecting these mainline oil and gas pipelines, oil and gas suppliers can avoid reducing the internal pressure and flow velocity of the pipelines, thereby avoiding a significant decrease in the transportation volume of oil and gas during pipeline internal inspection operations. Taking Central Asia–China and China–Myanmar natural gas pipelines as examples, the direct losses caused by the reduction in throughput during a single pipeline internal inspection operation can amount to hundreds of millions of dollars.

### 5.4. Support Vector Machine

**Figure 12:**
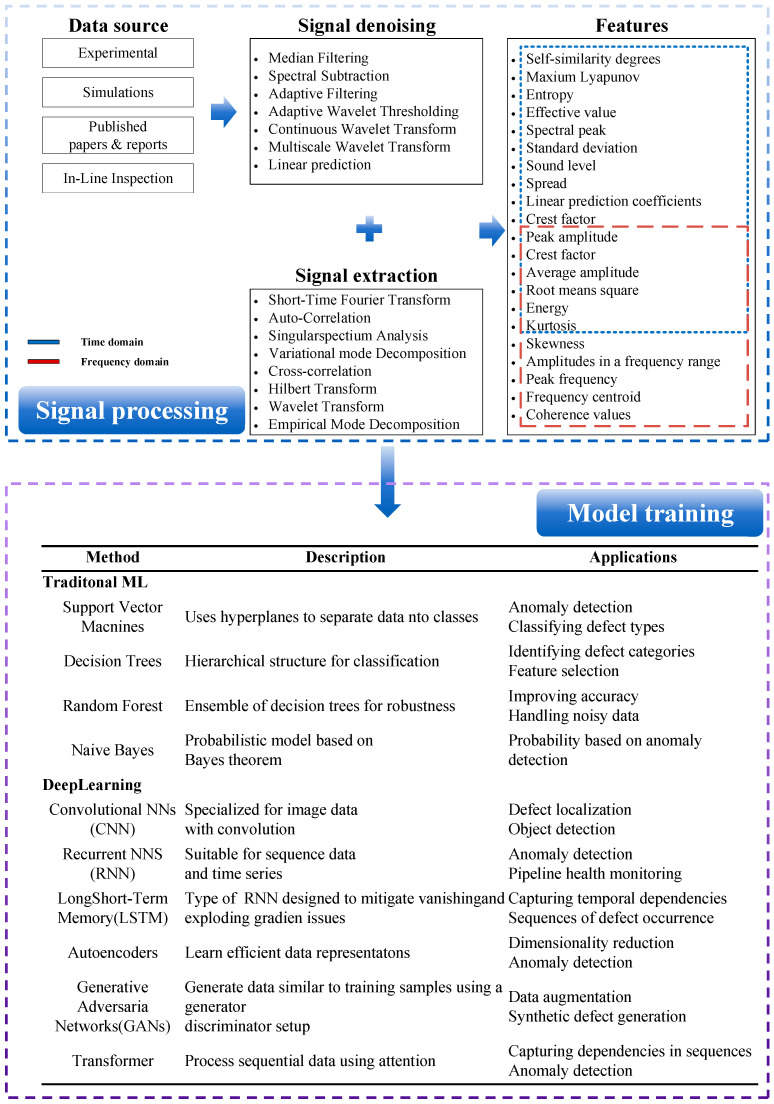
Ultrasonic Signal Analysis Workflow.

As the technology of ultrasonic internal inspection for pipelines continues to advance, the influx of a large amount of data imposes higher demands on signal processing methods. Signal processing of ultrasonic internal inspection signals can help reduce signal complexity and extract useful information [92,93]. Some common ultrasonic signal processing methods and data process flow are shown in Figure 12.

The support vector machine (SVM) is a supervised learning model used for classification and regression analysis. It has shown excellent performance in data mining and pattern recognition tasks in inspecting oil and gas pipelines. SVM can accurately handle linear problems; for complex nonlinear issues, data can be mapped to a high-dimensional space through nonlinear transformation functions [94,95,96]. To reduce computational costs, kernel functions can be used instead of nonlinear transformation functions [97]. Like SVM, other machine learning (ML) methods, such as decision trees, random forests, and naive Bayes have been used. These ML methods can extract useful features from acoustic signals and perform accurate classification and defect detection based on these features [98,99,100]. However, the class of traditional machine learning algorithms represented by support vector machines may not perform as well as deep learning when dealing with complex or large data models. They require manual feature engineering and may also rely on domain-specific expert knowledge. They are generally less suitable for handling high-dimensional data and nonlinear relationships. They are more suitable for small samples and nonlinear tasks but less suitable for large-scale datasets and multi-classification tasks [101,102,103].

Deep learning methods have gained significant attention with the increasing volume of data and the growing complexity of ultrasonic signals. Common deep learning networks include convolutional neural networks (CNN) [104], graph neural networks (GNNs) [98], recurrent neural networks (RNN) [105], long short-term memory (LSTM) [106], and AutoEncoder [107]. It is evident that data-driven techniques dominated by machine learning (ML) methods have demonstrated significant advantages in ultrasonic in-line inspection compared to physical models [108]. Their primary applications fall into the following three categories:

- Defect Classification:

Using deep learning algorithms to classify defects in the pipe body and weld area has been a prominent research topic in the ultrasonic inspection of oil and gas pipelines. Traditional defect classification methods struggle to differentiate between different types of defects accurately. Deep learning methods like CNN and LSTM often excel in handling complex signals, extracting temporal features, and improving detection accuracy. For example, Bettayeb et al. used wavelet transformation to extract feature vectors related to defects on the pipe body and weld, such as cracks, porosity, or inclusions. These feature vectors contain two-dimensional information about defects, and an artificial neural network (ANN) trained with a backpropagation algorithm was used to classify these feature vectors. The results showed that combining wavelet transformation and ANN could significantly suppress noise levels and improve defect classification accuracy [109]. Building on this, Sambath et al. analyzed 1084 samples using similar principles and achieved a reasonable classification rate of 94% [110]. Guo et al. proposed an image-based deep learning defect classification method. They used a gated recurrent unit fully convolutional network (GRU-FCN) to extract temporal features from A-scan ultrasound signals. The training, validation, and test datasets comprised 3600 ultrasound waveforms collected in experiments. The results were compared with LSTM, GRU, and ResNet, revealing that GRU-FCN achieved higher accuracy [111].

- Defect Characterization:

Defect characterization has been a critical basis for transitioning from non-destructive testing (NDT) to non-destructive evaluation (NDE). The accuracy of quantifying defects directly influences the effectiveness of non-destructive evaluation. C. Guo et al. proposed a novel residual vision transformer (Res-ViT) architecture based on deep residual networks (ResNet) and visual transformers (ViT). They conducted experiments on elliptical defects with inclination angles of 60° at different noise levels. Compared to the principal component analysis and nearest neighbor method, the root mean square error (RMSE) of the defect size was reduced by 61% [112]. Miorelli et al. introduced a CNN model for automatically locating and sizing defects from guided ultrasonic wave data. The deep learning model was trained using both simulated and experimental data, and the experiments demonstrated the model’s adaptability to real-world environments, achieving an accuracy of up to 90% [113]. Bai et al. compared the classical Bayesian inversion method proposed by Miorelli and a CNN regression model. In this study, the classical Bayesian method exhibited higher accuracy and lower uncertainty in defect characterization, but it introduced more discreteness due to the model’s inherent uncertainty [114].

- Data Preprocessing:

In practical experiments or industrial applications, data often come with noise due to environmental factors or equipment characteristics, which can affect subsequent signal analysis. Data preprocessing aims to improve the quality of ultrasonic detection data. Data preprocessing techniques include but are not limited to image denoising, feature recognition and extraction, and data compression. Noise reduction primarily aims to improve the signal-to-noise ratio (SNR). Yang et al. designed a lightweight denoising network called the global interactive attention lightweight denoising network (GIALDN) for analyzing vibration signals and locating internal defects in CFRP laminates. In GIALDN, a threshold-based denoising method was used to eliminate noise-related features and enhance feature discriminability. The results showed that GIALDN achieved a location accuracy of 98.68%, which was more than 15% higher than VGGnet11 and FaultNet, and outperformed LSTM, RNN, Rsenet18, SEresnet18, and Densenet121 [115]. For ultrasonic defect detection, which typically involves a larger volume of data compared to conventional eddy current testing, compressing input data into latent features can replace the indiscriminate retention of raw data. Kesharaju proposed a feature selection method based on the genetic algorithm (GA) and fully convolutional neural network (FCNN). This method used a subset of preselected features as input to the FCNN model. The results showed that the performance improved by 94% compared to the model based on principal component analysis (PCA) [116].

**Limitations:** Currently, most machine learning methods are primarily in the theoretical validation stage, with only a few models being applied in industrial practices, and their performance is not ideal. The main challenge lies in the difficulty of obtaining a sufficient amount of high-quality data due to the confidentiality and sensitivity of oil and gas pipeline data. Therefore, most research based on machine learning methods is conducted using simulated data or laboratory data for defect or anomaly detection analysis, making it challenging to adapt to real-world pipeline inspection data.

## 6. Potential Challenges and Opportunities

Considering the current testing technology, it is evident that we are still in the early stages of ultrasonic non-destructive evaluation. Therefore, in the foreseeable future, internal ultrasonic inspection of oil and gas pipelines will continue to face the following challenges and opportunities:

(1) Exploration of novel sensor designs and materials for enhanced sensitivity and resolution in UT inspections.
(2) Research into multi-modal sensor arrays combining UT with other NDT techniques (e.g., electromagnetic acoustic transducers or distributed fiber optic sensors) for complementary defect characterization.
(3) Miniaturization of sensors for improved accessibility to challenging pipeline geometries and locations.
(4) Addressing the impact of velocity and vibration on the accuracy and precision of detection from both theoretical and sensor optimization perspectives to achieve ultrasonic inspection at the velocity of the conveying medium.
(5) Development of novel encoding algorithms to mitigate artifacts and enhance the sensitivity of ultrasound imaging.
(6) Air-coupled testing holds promising applications in the inspection of oil and gas pipelines because of its ability to detect cracks and metal loss defects in thick-walled pipelines that magnetic flux leakage testing may not detect.
(7) Integration of machine learning algorithms for optimized encoding parameter selection, real-time adaptive imaging and corrosion quantification [117].
(8) Exploration of novel materials with tailored acoustic properties for matching layers to optimize acoustic impedance matching and minimize signal loss at transducer interfaces.
(9) The enhancement of generalization and transfer learning capabilities of ultrasonic data analysis models established based on simulation and laboratory in industrial application environments.

## 7. Conclusions

In this study, we conducted a bibliometric analysis based on 350 ultrasonic testing-related publications from the Web of Science (WOS) database since 1992. Utilizing data visualization techniques, we identified the most influential countries, institutions, and publications in the field globally. Our analysis of the data level elucidates the potential developmental reasons behind these trends. We observed that Western developed countries, represented primarily by the UK and the USA, continue to maintain a central position in the realm of ultrasonic testing. Due to industrial development needs, China has shown rapid progress in ultrasonics in recent years, and the focal point of ultrasonic testing is progressively shifting from theoretical aspects to practical scene applications.

To mitigate the impact of personal biases on this research, we relied on cluster analysis and timeline methods to pinpoint the current research hotspots in ultrasonic testing for oil and gas pipelines. This approach enabled us to delineate the research tasks in various stages of oil and gas pipeline ultrasonic testing. Building on the hotspot analysis, we also presented the ongoing opportunities and challenges in this field.

Like ultrasonic testing technology itself, which possesses unique advantages and limitations, our study is not without its constraints. For instance, due to the limitations of the database, not all relevant literature in the field may be covered. However, the data sourced from WOS ensures a comprehensive collection and analysis of core research findings in the ultrasonic testing domain.

## Figures and Tables

**Table 1:** The Most Influential Countries in the Global Ultrasonic Field (Institutions).

Country	Institutions	Burst	Centrality
China	61	3.26	0.36
England	48	0	0.73
Germany	33	2.1	0.27
USA	32	1.78	0.9
Italy	27	0	0.63
Republic of Korea	23	0	0.34
Japan	17	2.08	0.16
Brazil	14	0	0.12
India	13	1.87	0.19
France	12	2.44	0.22

**Table 2:** Global Influential Countries in the Ultrasonic Field Based on Different Criteria.

Standard	Institutions	Burst	Centrality
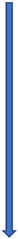	China	China	USA
	England	France	England
	Germany	Germany	Italy
	USA	Japan	China
	Italy	India	Republic of Korea
	Republic of Korea	USA	Germany
	Japan	England	France
	Brazil	Italy	India
	India	Republic of Korea	Japan
	France	Brazil	Brazil

**Table 3:** The Most Influential Institutions in the Global Ultrasonic Field.

Institutions	Total Link Strength	Documents	Citations	Country
University of Warwick	12	7	334	UK
Federal Institute for Materials Research and Testing	14	4	207	Germany
Seoul National University	4	3	185	Republic of Korea
Newcastle University	16	6	138	UK
University of Palermo	34	6	127	Italy
Kaunas University of Technology	6	9	119	Lithuania
Brunel University London	30	8	118	UK
University of Electronic Science and Technology of China	9	6	108	China
Cranfield University	5	3	92	UK
City University of Hong Kong	16	6	90	China

**Table 4:** The Most Influential Journals in the Global Ultrasonic Field.

Journals	Total Link Strength	Documents	Citations
*NDT & E International*	220	49	2055
*Sensors*	199	64	618
*Ultrasonics*	123	75	715
*Insight*	106	56	709
*Measurement*	94	37	680
*Structural Health Monitoring*	82	32	440
*Applied Sciences-Basel*	74	26	478
*Journal of Nondestructive Evaluation*	74	22	347
*Optik*	72	21	301
*Applied Physics A-Materials* *Science & Processing*	58	16	365

**Table 5:** Common Defect Types and Causes.

Type	Kind		Cause	Location
Planar defect	Crack	Fatigue Crack	Fatigue cracks are cracks formed in pipes under the action of alternating or cyclical loads, such as tension, compression, or bending.	Weld & Body
		Stress Corrosion Cracking (SCC)	Stress corrosion cracks are formed in pipelines under the simultaneous influence of stress and environmental corrosion.	
		Hydrogen-Induced Cracking (HIC)	Metals absorb hydrogen atoms. When subjected to stress, hydrogen causes a change in the material’s atomic structure, making it more susceptible to fracture.	
	Infusion		During welding, incomplete fusion occurs when the welding material fails to fully melt and fuse with the base material. This can result in the presence of cracks or unjoined areas in the weld, thereby reducing the strength and reliability of the weld.	Weld
	Underpenetration		Incomplete penetration occurs when welding material fails to fully penetrate through to the base material during welding. This results in the presence of gaps in the weld, which may create weak points, especially under pressure loads.	Weld
	Undercut		The arc melts the edge of the base material at the weld seam, leaving a groove. Undercut weakens the load-bearing cross-section of the joint, making it susceptible to stress concentration.	Weld
Volumetric defect	Porosity		During the welding process, gas does not escape in time and forms voids inside or on the surface of the weld metal.	Weld & Body
	Inclusion		Inclusions or foreign substances present in the interior of the weld metal or fusion line.	Weld & Body
	Corrosion		Pipeline damage caused by chemical environmental corrosion or stray currents affecting the material.	Weld & Body
	Dent			Body

**Table 6:** Types of Internal Inspectors and Detection Capabilities.

Type	Kind		MFL	UTWM ^a^	UTCD ^a^	EMAT	Geometric Inspection	EC
Planar defect	Crack	Fatigue Crack	×	×	√+	√+	×	√−
		Stress Corrosion Cracking (SCC)	×	×	√+	√+	×	√−
		Hydrogen-Induced Cracking (HIC)	×	×	√+	×	×	√−
	Infusion	Straight Weld	×	×	√+	√+	×	√
		Spiral Weld	√+	×	×	×	×	√
		Girth Weld	√+	×	√+	×	×	×
	Underpenetration		√	√	√	√	×	√
	Undercut		×		√	√	×	√
Volumetric defect	Porosity		√	×	√+	×	×	×
	Inclusion		√−	√+	√−	√−	×	×
	Corrosion	Internal	√+	√+	×	×	√−	√−
		External	√+	√+	×	×	×	×
Geometry	Dent		√	√	×	×	√+	×
	Distortion		√	√	×	×	√+	×

^a^: Only applied in liquid environment; ×: Undetectable; √: Detectable; √−: Detectable with Limitations; √+: Detectable and Quantifiable.

**Table 7:** Comparison of Nondestructive Testing Methods for Surface Defects.

	Penetration Testing	Magnetic Particle Testing	Eddy Current Testing
Principle	Capillary phenomenon	Magnetic force	Electromagnetic induction
Range	Any non-porous material	Ferromagnetic materials	Conductive materials
Position	surface opening defects	surface or near-surface defects	Surface
Sensitivity	High	High	Low
Speed	Slow	Fast	Fast, can be automated
Effect of Defect Orientation on Detection Probability	Unaffected by defect orientation	Affected by defect orientation, easily detects defects perpendicular to the direction of magnetic lines	Affected by defect orientation, easily detects defects perpendicular to the direction of eddy currents
Effect of Surface Roughness on Detection Probability	Rougher the surface, lower the probability of detection	Affected, but less than penetration testing	Greatly affected

**Table 8:** Pipeline External Inspection Methods Without Excavation.

Method	Coating Delamination	Size	Location	Severity	External Interference	Cathodic Protection Evaluation	Operator	Pipeline Inspection	Current Source
Pipeline-to-Soil Potential Survey (P/S)	✕	✕	✕	✕	✓	✓	✓	✓	GP/G
Close-Interval Potential Survey (CIPS)	✕	✕	✕	✓	✓	✓	✓	✓	GP/G
Direct Current Voltage Gradient (DCVG)	✕	✓	✓	✓	✕	✕	✓	✓	D
Pearson	✕	✓	✓	✕	✓	✓	✓	✓	A
Pipe Current Attenuation Method	✕	✕	✓	✕	✓	✓	✓	✓	A
Frequency Domain Reflectometry (FDR)	✕	✕	✕	✕	✓	✓	✓	✓	A

Cathodic Protection System—GP. Direct Current—D. Alternating Current—A. Ground—G.

**Table 9:** Comparison of Non-Contact Ultrasound-Guided Wave Techniques.

Technology Type	Principle of Detection	Advantages	Limitations
Laser Ultrasonic-Guided Wave Testing	Utilizes laser pulses to induce stress pulses in the test piece through thermoelastic or ablation effects, generating ultrasonic wave signals.	Non-contact detection suitable for high-temperature, high-pressure, toxic environments; high sensitivity allows for inspection on complex structures.	Efficiency of laser conversion to ultrasonic signals may be low; Sigh-power lasers may damage the surface of the specimen; sensitivity of detection may be suboptimal.
Electromagnetic Ultrasonic-Guided Wave Testing	Employs a probe to emit ultrasonic-guided waves, using the time difference of reflections from the inner and outer walls of the pipeline to determine wall thickness and corrosion.	Capable of long-distance detection, convenient operation, and minimally affected by external factors, such as temperature, pressure, and internal flow media.	Direct measurement of wall thickness is not possible; sensitive to defects in wall depth and circumferential width, only axial length of defects can be measured within a certain range.
Air Coupled Ultrasonic-Guided Wave Testing	Uses air as the coupling medium, transmitting and receiving ultrasonic waves through air-coupled transducers to detect material defects.	Non-contact detection without the need for a coupling agent, suitable for high-temperature or inaccessible environments; capable of inspecting very thin workpieces.	Due to the attenuation of ultrasound by air and impedance differences at the air–solid interface, there is significant reflection and low conversion efficiency of ultrasonic waves, resulting in a potentially low SNR.

**Table 10:** Types of Internal Inspectors and Inspection Capabilities.

Corporation	Product	Crack (Length × Depth)	Operation Speed	Wall Thickness (mm)	Depth Sizing	Length Sizing	Medium Type	Orientation to Pipe Axis	Min. Bend Radius
P II	UltraScan CD EDGE	15 × 1	≤2.5 m/s	5~10	±0.7	±7.5	liquid	0°	1.5 D
	UltraScan CDP/DUO	25 × 1	≤5 m/s	5~13	±0.7	±7.5	liquid	0°	1.5 D
	EmatScan CD	50 × 2	≤2.5 m/s	7~13	±1.1	±10	G/L	0°	1.5 D
ROSEN	ROCDeMAT-c	40 × 2	≤2.5 m/s	0~20 mm	±0.15 t	±20	G/L	±18°	1.5 D
TDW	SpirALL	30 × 2	≤2.5 m/s	0~13 mm	±1	±10	G/L	±10°	1.5 D
CNPC	/	50 × 2	≤2.5 m/s	7~13	±1.1	±10	liquid	0°	1.5 D
NDT Global	PROTON	20 × 1	≤1.4 m/s	7~13	±1	±10	liquid	±10°	3 D
	EVO Eclipse UCx	20 × 1	≤4 m/s	0~13 mm	±1	±10	liquid	±10°	1.5 D
